# Genomic analysis of SARS-CoV-2 reveals local viral evolution in Ghana

**DOI:** 10.1177/1535370220975351

**Published:** 2020-12-16

**Authors:** Joyce M Ngoi, Peter K Quashie, Collins M Morang'a, Joseph HK Bonney, Dominic SY Amuzu, Selassie Kumordjie, Ivy A Asante, Evelyn Y Bonney, Miriam Eshun, Linda Boatemaa, Vanessa Magnusen, Erasmus N Kotey, Nicaise T Ndam, Frederick Tei-Maya, Augustina K Arjarquah, Evangeline Obodai, Isaac D Otchere, Yaw Bediako, Joe K Mutungi, Lucas N Amenga-Etego, John K Odoom, Abraham K Anang, George B Kyei, Bright Adu, William K Ampofo, Gordon A Awandare

**Affiliations:** 1 West African Centre for Cell Biology of Infectious Pathogens, College of Basic and Applied Sciences, University of Ghana, Accra, GH 0233, Ghana; 2 Noguchi Memorial Institute for Medical Research, College of Health Sciences, University of Ghana, Accra, GH 0233, Ghana; 3 Department of Biochemistry, Cell and Molecular Biology, School of Biological Sciences, University of Ghana, Accra, GH 0233, Ghana; 4 The Francis Crick Institute, 1 Midland Road, London NW1 1AT, UK; 5 Mère et Enfant en Milieu Tropical, Institut de Recherche pour le Développement, Université de Paris, Paris F-75006, France; 6 University of Ghana Medical Centre, University of Ghana, Accra, GH 0233, Ghana

**Keywords:** SARS-CoV-2, COVID-19, novel coronavirus, genomics, evolution

## Abstract

The confirmed case fatality rate for the coronavirus disease 2019 (COVID-19) in Ghana has dropped from a peak of 2% in March to be consistently below 1% since May 2020. Globally, case fatality rates have been linked to the strains/clades of circulating severe acute respiratory syndrome coronavirus 2 (SARS-CoV-2) within a specific country. Here we present 46 whole genomes of SARS-CoV-2 circulating in Ghana, from two separate sequencing batches: 15 isolates from the early epidemic (March 12–April 1 2020) and 31 from later time-points ( 25–27 May 2020). Sequencing was carried out on an Illumina MiSeq system following an amplicon-based enrichment for SARS-CoV-2 cDNA. After genome assembly and quality control processes, phylogenetic analysis showed that the first batch of 15 genomes clustered into five clades: 19A, 19B, 20A, 20B, and 20C, whereas the second batch of 31 genomes clustered to only three clades 19B, 20A, and 20B. The imported cases (6/46) mapped to circulating viruses in their countries of origin, namely, India, Hungary, Norway, the United Kingdom, and the United States of America. All genomes mapped to the original Wuhan strain with high similarity (99.5–99.8%). All imported strains mapped to the European superclade A, whereas 5/9 locally infected individuals harbored the B4 clade, from the East Asian superclade B. Ghana appears to have 19B and 20B as the two largest circulating clades based on our sequence analyses. In line with global reports, the D614G linked viruses seem to be predominating. Comparison of Ghanaian SARS-CoV-2 genomes with global genomes indicates that Ghanaian strains have not diverged significantly from circulating strains commonly imported into Africa. The low level of diversity in our genomes may indicate lower levels of transmission, even for D614G viruses, which is consistent with the relatively low levels of infection reported in Ghana.

## Impact statement

This report presents the most in-depth analysis of multiple SARS-CoV-2 whole genomes in Africa. The results provide new insights about the level of viral importation, local transmission dynamics, and viral evolution. We have identified that by June 2020, two major clades, 19B/S/B4 and 20B/GR/A2a, were likely responsible for ∼86% of all COVID-19 cases in Ghana, and that the Accra suburb Ayawaso is a hotbed of transmission. Our results suggest that swift containment measures likely limited the diversity of viruses available to seed the Ghanaian COVID-19 outbreak. Significantly, our data suggest that although spike substitution D614G may enhance transmission, its role in enhancing disease pathogenesis remains unclear. The whole viral genomes reported here were amongst the first generated in Africa and demonstrate the progress made in building local capacity for performing high-quality molecular epidemiology studies. This work establishes a reference framework for future molecular studies of viral transmissions, as African countries gradually reopen their borders.

## Introduction

The coronavirus disease 2019 (COVID-19) pandemic, caused by severe acute respiratory syndrome coronavirus 2 (SARS-CoV-2) is a growing public health nightmare with over 30 million individuals infected as at 20 September 2020 and over 950,000 deaths worldwide (Table 1).^1,2^ There is no approved standard treatment, cure, or vaccine against COVID-19 and it is expected that both infections and mortalities will continue to rise globally.^3–5^ The epicenter of the pandemic has moved from Wuhan, China, through Europe and is now in the United States of America where currently the highest number of infections and deaths is being reported (World Health Organization.^2^ At all the epicenters, an initial slow infection rate was followed by a rapid exponential infection phase possibly associated with factors including socio-demographic profiles, host immune responses, and viral genetics.^6^ The virus SARS-CoV-2 was first detected in Africa on 14 February 2020, in Egypt and has subsequently spread to every country on the continent.^7–9^ As of 20 September 2020, there were 1,145,397 cumulative cases and 24,757 cumulative deaths in Africa.^2^

**Table 1.** Comparison of Ghanaian outbreak with similarly timed outbreaks of the COVID-19.

**Country**	**Continent**	**Date of 1st confirmed case**	**Days ahead of 1st Confirmed Ghana case**	**Lockdown details**	**Situation Update**					
					**1 April 2020**			**20 September 2020**		
					**Cases**	**Deaths**	**CFR (%)**	**Cases**	**Deaths**	**CFR (%)**
World	World	31-Dec-19	72	Varied	823,626	40,598	4.93	30,675,675	954,417	3.11
Ghana	Africa	12-Mar-20	0	3 wks 30 March – 20April	152	5	3.29	45,877	297	0.65
South Africa	Africa	5-Mar-20	7	5 wks 26 Mar – 16 Apr + 16th – 30th Apr	1353	5	0.37	659,656	15,940	2.42
Kenya	Africa	13-Mar-20	−1	3 wks 23rd Apr – 6th July	50	1	2.00	36,829	646	1.75
Cote D'Ivoire	Africa	11-Mar-20	1	2 months 27th March-	169	0	0.00	19,200	120	0.63
Morocco	Africa	2-Mar-20	11	20th March – 24 June	638	36	5.64	99,816	1795	1.80
Uzbekistan	Asia	15-Mar-20	−3	23rd Mar- May + 10th July – 1 Aug	173	2	1.16	51,235	429	0.84
Peru	South America	6-Mar-20	6	106 days 16th March – 31st June	1065	24	2.25	756,412	31,283	4.14
Bulgaria	Europe	7-Mar-20	5	13th March – 15th June	399	8	2.01	18,819	755	4.01
Mongolia	Asia	10-Mar-20	2	6 days 10th March-	12	0	0.00	311	0	0.00
Turkey	Europe	11-Mar-20	1	23rd May – 1st June	13,531	214	1.58	301,348	7445	2.47
Switzerland	Europe	5-Mar-20	7	15-Jun	16,108	373	2.32	49,171	1764	3.59
Palestine	Asia	5-Mar-20	7	Varied for different parts	134	1	0.75	44,763	291	0.65
Uruguay	South America	13-Mar-20	−1	13th March – May	320	1	0.31	1890	46	2.43

Note: The table was generated from 1 April 2020 and 20 September 2020, World Health Organization (WHO) situational reports.^1,2^

CFR: Case fatality ratio (number of COVID-19 deaths/number of confirmed COVID-19 cases × 100.

SARS-CoV-2 is highly infectious and appears to have a relatively stable genome. However, sites of selective mutations have been identified and strains from specific geographical areas may differ.^10–12^ It is, however, not known whether the selective mutations result in specific strains that are more adapted for high transmission in different populations. Such questions may be answered as more data become available through the ongoing concerted global effort to generate more SARS-CoV-2 genome data which would also inform drug discovery, vaccine design, and COVID-19 transmission dynamics worldwide. Since the first complete genome of the virus from Wuhan was published in late 2019, whole genomes of several strains across the globe have been added to the public databases such as GISAID and GENBANK.^13^ Nonetheless, SARS-CoV-2 genome sequence data from COVID-19 cases in sub-Saharan Africa currently constitute less than 2% of sequences in genome repositories.^14^ It is uncertain whether Africa will become the next epicenter of COVID-19; however, timely high-quality SARS-CoV-2 genome data may help inform the dynamics of the disease spread for better control measures. With the relatively higher infectious disease burden in sub-Saharan Africa, it is possible that potentially cross-reactive pre-existing immunity may drive a stronger immune selection that could affect the virus evolution in such populations.^15^

Cases of COVID-19 in Ghana appeared to be largely asymptomatic with a low reported case fatality ratio (CFR) below 0.64% as at 23 September 2020.^16^ This pattern of mild clinical presentations of COVID-19 appears consistent across Africa, and therefore a comprehensive genomic analysis of the SARS-CoV-2 strains circulating on the continent is required to determine the role of viral evolution in determining disease severity.^14,17^ Thus far, SARS-CoV-2 genomic data are available from Nigeria, Senegal, Kenya, and Ghana; however, comprehensive analysis of these data have not been published.^14,18,19^

In this report, we present a comprehensive molecular epidemiological analysis of SARS CoV-2 genomic data from 46 PCR-confirmed COVID-19 cases in Ghana. These cases included 15 from the beginning of the epidemic in Ghana in March 2020, and 31 additional cases from the subsequent two months as the outbreak spread in Ghana. The data provide both valuable information about the SARS-CoV-2 clades circulating in Ghana and the prevalence of mutations in key viral genes, and thus provide a vital reference framework for monitoring the evolution of the virus as the pandemic spreads in Africa.

## Materials and methods

### Study design

Samples analyzed in this study were selected at two time points from the biorepository at the Noguchi Memorial Institute for Medical Research, University of Ghana, which is the designated national reference laboratory for testing suspected COVID-19 cases. The biorepository includes samples from both primary cases and their close-contacts. The study was approved by both the Ethics Review Committee of the Ghana Health Service and the Ethical Committee of the College of Basic and Applied Sciences (University of Ghana). A simplified flow chart of experimental and data analysis procedures is shown in Supplementary Figure 1.

### Sample processing

The QIAamp viral RNA extraction kit (Qiagen, Hilden, Germany) was used to extract total RNA from nasopharyngeal and oropharyngeal samples which had previously been confirmed as SARS-COV-2 positive by real-time RT-PCR. Samples that were chosen for sequencing had cycle threshold (C_T_) values in the range 18–35; a C_T_ value below 40 was considered a positive test result. The extracted total RNA concentration was measured using Qubit™ RNA HS Assay Kit on a Qubit 4 Fluorometer (ThermoFisher Scientific™, MA USA). The integrity and quality of RNA were checked using the Agilent RNA 6000 Nano Kit on the Bioanalyzer (Agilent™ Tech. Inc. CA USA). The extracted RNA was either immediately used (first batch) or stored at −80°C until cDNA synthesis (second batch).

### Illumina MiSeq sequencing

Complementary DNA (cDNA) was prepared from the extracted RNA using SuperScript™ IV VILO™ (SSIV VILO) Master Mix (ThermoFisher Scientific MA USA). The cDNA was subjected to a multiplex PCR using the ARTIC nCoV-2019/V1 (first batch) or V3 (second batch) primers as per the protocol^20^ (Josh Quick, 2020). The PCR products were visualized for the presence of 400 bp fragments and then purified using 1× Agencourt AMPure XP (Beckman Coulter Inc., TX USA). Products were quantified using the Qubit™ dsDNA HS Assay Kit (ThermoFisher Scientific, MA USA) and concentrations normalized to 1 ng. Libraries for sequencing were prepared from the normalized products using the Nextera XT DNA Library Preparation Kit and the Nextera XT Index Kit v2 Set-A (Illumina) according to manufacturer’s instructions. Each barcoded library was then purified using Agencourt AMPure XP beads (Beckman Coulter Inc., TX USA) and thereafter the size distribution and library quality control carried out using the Agilent 2100 Bioanalyzer. The purified libraries were quantified using the Qubit™ dsDNA HS Assay Kit on the Qubit 4.0 fluorometer (ThermoFisher Scientific, MA USA) and normalized to equimolar concentrations. A pool of all the normalized libraries was prepared and diluted to a final concentration of 10pM, spiked with 5% PhiX, and sequenced on the Illumina MiSeq system using the MiSeq® Reagent Kit v3 600 cycle.

### Generation of SARS-CoV-2 genomes

A Nextflow pipeline that automates the ARTIC network nCoV-2019 novel coronavirus bioinformatics protocol was used in assembling the Illumina sequencing data into consensus genomes.^21^ SARS-CoV-2 sequencing read quality was visualized using fastQC (version 0.11.9) and trimmed using trim galore (Version 0.6.5). BWA (version 0.7.17)^22^ was used to map trimmed FastQ reads to an indexed SARS-CoV-2 reference genome (MN908947.3). The mapped genomes were converted to BAM, sorted, and indexed using samtools (Version 1.9). iVar (version 1.0) was used for primer trimming, variant calling, and consensus generation.^23^ Primer sequences (arctic primers V3) were trimmed using iVar with the following criteria: reads without primer sequences were allowed, retaining a minimum of 20 reads after trimming, and the sliding window quality threshold for keeping reads after primer trimming was 20. Variants were generated using the following criteria: minimum samtools mpileup depth of 100,000 reads, 10 reads minimum coverage depth to call variant, iVar minimum map quality to call variant was 20, and iVar frequency threshold to call variant was 0.25. Consensus genomes were generated using similar mpileup depth, frequency threshold, and minimum map quality. Assembled genomes were subjected to final QC and deposited in the GenBank database with sample IDs MT890204–MT890249.

### Phylogenetic analysis

A coverage map was generated by comparing the Ghanaian SARS-CoV-2 genomes and the reference genome (Wuhan-Hu-1/2019) using Nextclade (version 0.7.5).^24^ Nextclade is a web-based tool which performs banded Smith–Waterman alignment with an affine gap-penalty. We performed basic phylogenetic analysis on SARS-CoV-2 genomic data using Nextstrain pipelines.^24^ The pipeline incorporates Augur for generation of phylogenetic tree and Auspice for visualizations. Briefly, we filtered the sequences depending on the quality of alignment to the SARS-CoV-2 reference genome (MN908747.3), and masked 100 bp from start and 50 bp at the end, as well as regions prone to sequencing errors (13402, 24389 and 24390).^24^

The initial maximum likelihood phylogenetic tree was constructed using a fast and stochastic algorithm (IQ-TREE) and a generalized time reversible (GTR) substitution model.^25^ First, we generated a rooted time-resolved maximum likelihood phylogenetic tree, whereby the branch lengths on the tree represent time of sample collection. The tree was constructed using a pre-existing phylogenetic tree, a sequence alignment, and the metadata information that had the sample collection dates. Then, we generated a maximum likelihood phylogenetic unrooted tree which showed clade assignments and clustering of the samples. We also performed clade analysis to determine the dominant clades in the Ghanaian population and then determined the divergence of the SARS-CoV-2 genomes in Ghana relative to the Wuhan reference genome (Wuhan/Hu-1/2019). To generate a divergence phylogenetic tree, we used the following parameters; clock rate of 0.0008, a clock standard deviation of 0.0004, and mutations as the divergence unit.

## Results

### Patient characteristics

Ghana started screening for SARS-CoV-2 at the ports of entry on 6 February 2020, even before COVID-19 was declared a “Pandemic” on 11 March 2020 by the World Health Organisation.^26–28^ Ghana had implemented monitoring controls at all major ports of entry and suspected cases reporting to health facilities were forwarded to the Noguchi Memorial Institute for Medical Research (NMIMR) for testing. This yielded the first two confirmed COVID-19 positive cases on 12 March 2020.^29^

As of 1 April 2020, there were 195 confirmed cases in Ghana, as such our first batch of samples (20/195) represented ∼10% of total confirmed cases and were therefore a fair representation of the virus circulating at the time. Our second batch of samples (*n* = 36) were taken two months after the first batch, to evaluate how the virus had evolved over this period. The first batch of samples had mostly been obtained from the Greater Accra Region. The second batch included a few samples from the Western and Central Regions, which only had their first cases after the first batch of sequencing had been done. Consistent with the general observation in COVID-19 cases in Ghana, elevated body temperature was not a common characteristic of our cohort, with only two patients showing temperatures above 37.4°C at the time of sample collection (Supplementary Table 1). Data on age were available for majority of cases (*n* = 33/46), and the range was 4–63 years, with the plurality being between 20–36 years. The cases (*n* = 41/46) with sex data were also predominantly male (58.5%, 24/41), consistent with infection patterns observed globally (Supplementary Table 1).

### Sequencing and genome assembly

**Figure 1.**
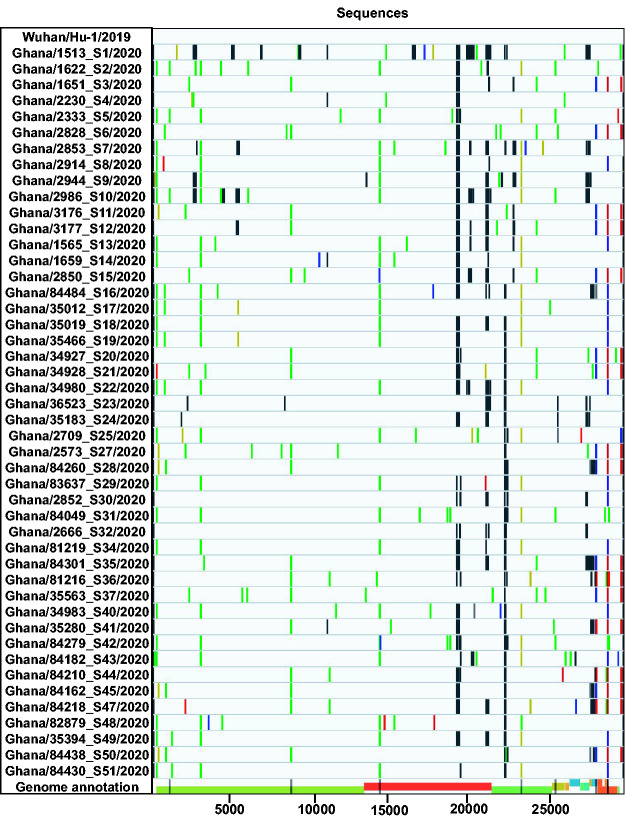
Genomic map showing coverage and homology of Ghanaian SARS-CoV-2 sequences to the Wuhan/Hu-1/2019 genome. The grey colors indicate Ns and gaps, while the rest of the colors indicate differences between sequences without any particular order. We used Nextclade—a webtool that identifies the differences between sequences such as mutations, Ns, and gaps. Nextclade uses an algorithm which estimates the alignment of each genome to the reference genome using short 21-mers along the sequence thereby performing a banded Smith–Waterman alignment with an affine gap-penalty. (A color version of this figure is available in the online journal.)

Although the ARTIC protocols were originally optimized for use on nanopore devices, we successfully adapted them for Illumina MiSeq (Supplementary Figure 2). Samples with cycle threshold (Ct) lower than 25 were more likely to be successfully amplified. In total, we obtained 46/56 full-length genomes from sequencing 20 samples in our first batch and 36 samples in our second batch. All the SARS-CoV-2 genomes were confirmed to be variants of the beta-coronavirus SARS-CoV-2, the causative organism of COVID-19, and mapped to the reference strain from Wuhan with at least 99.8% similarity and over 91% sequence coverage (45/46 genomes had >95% coverage) (Figure 1). Genomes were of high quality with nucleotide ambiguity represented by N being < 3% in majority of the genomes (39/46) (Figure 1). Only 8/46 genomes had N% higher than 2.5% and of these only 1 had N% >6% (Ghana/1513_S1/2020: N%»9%). The change from using the ARTIC nCoV-2019/V1 in the first batch of sequencing to ARTIC nCoV-2019/V3 primer set in the second batch improved minimum sequence coverage from 91% to 96% (Figure 1).

### Phylogenetic analysis

**Figure 2.**
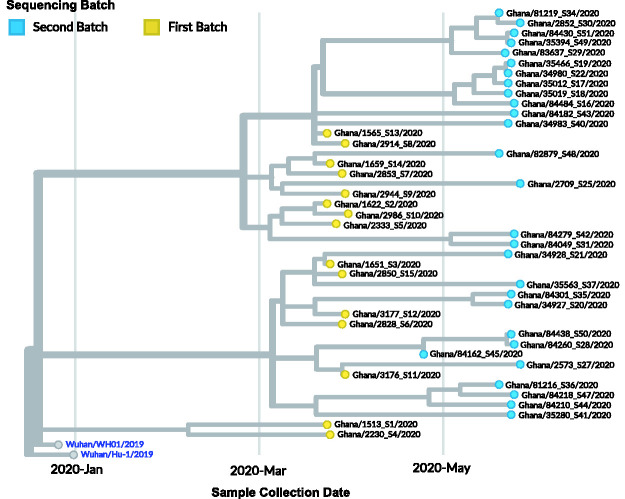
Rooted time-resolved maximum likelihood phylogenetic tree. The branch lengths on the phylogenetic tree represent calendar time of sample collection; 15/20 samples were collected March – April (First Batch), while the 31/36 were collected late in May (Second Batch). There is high level of relatedness among genomes sequenced at two different calendar times. Majority of the second batch samples (12/36) branch out from Ghana/1565_S13/2020 and Ghana/2914_S8/2020. Several others (8/36) are phylogenetically related to Ghana/3176_S11/2020. The initial maximum likelihood phylogenetic tree was constructed using a fast and stochastic algorithm (IQ-TREE) and a generalized time reversible (GTR) substitution model, then modified using auger (refine) time tree option. The tree is rooted to the Wuhan reference genome (Wuhan/Hu-1/2019). (A color version of this figure is available in the online journal.)

**Figure 3.**
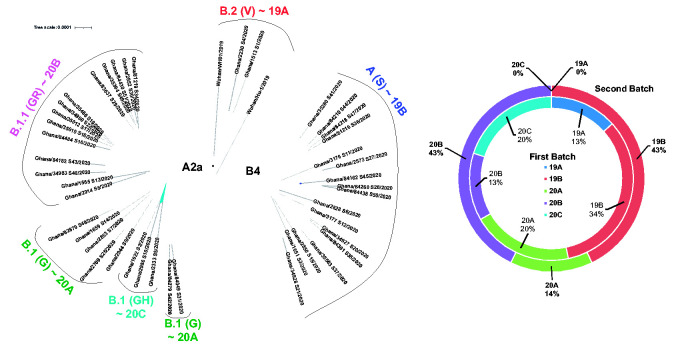
Circulating clades of SARS-CoV-2. (a) The tree indicates the clades circulating in Ghana (*n* = 43/46) whereby majority of the samples belong to clade 19B/S/B4 and clade 20A,B,C/G,GH,GR/A2a. Two samples had mutations that ensure they remained in the 19A/V clade, hence cluster with the Wuhan genomes. The 43 samples can also be clustered into two major legacy clades, the A2a and B4 whereby the B4 clade comprises of the pangolin clade A, while the A2a clade comprises samples in the B.1 and B.1.1 pangolin clades. The first clade to emerge out of Wuhan was 19A which quickly diverged into 19B, which has clearly dominated the samples that were sequenced in Ghana;20A emerged from 19A and dominated the European outbreak in March whereby it spread to Ghana by travelers; 20B and 20C are just genetically distinct sub-clades 20A. (b). Clade composition of sequenced viral isolates shows growing domination of 19B in the samples that were sequenced. (A color version of this figure is available in the online journal.)

Phylogenetic analysis on SARS-CoV-2 using the Nextstrain pipelines showed time-resolved phylogenies of samples from the first and second sequencing batches (Figure 2). The viruses isolated from these two batches were closely related, with a disproportionate number of the second batch samples (12/31) branching out from Ghana/1565_S13/2020 and Ghana/2914_S8/2020. Several others (8/31) were phylogenetically related to Ghana/3176_S11/2020 (Figure 2). We utilized three main nomenclature codes for clade analysis based on the evolving expert consensus represented on the GISAID website for SARS-CoV-2; Legacy clade assignment based on phenotypic variation^30^ and ‘Nextstrain clade’ assignment based on nucleotide polymorphisms. However, the GISAID clade assignment takes both approaches into account. Clades could be fully assigned in 41/46 genomes regardless of assignment method; others were “unassigned” (Supplementary Table 2). The first batch of 15 sequences clustered to several clades (nomenclature-GISAID/Legacy/Nextstrain): 13% 19 A/V/undetermined, 34% 19B/S/B4, 20% 20 A/G/A2a, and 13% 20B/GR/A2a and 20% 20 C/GH/A2a (Figure 3(a) and (b)). The imported cases mapped to circulating viruses in their country of origin, namely, Norway, United Kingdom, India, Hungary, and the United States. All imported strains mapped to the European super-clade A within the L lineage, whereas 5/9 locally infected individuals harbored the legacy B4 clade, from the East Asian superclade B (S-lineage) (Figure 3, Supplementary Figure 3).

The second batch of genomes showed much less clade diversity than the first batch, but more mutational diversity. Forty-three percent (43%) of genomes clustered to 19B/S/B4, 14% to 20 A (7% to 20 A/G/A2a, 7% to 20 A/GH/A2a), and 43% clustered to 20B/GR/A2a (Figure 3(b), Supplementary Table 2). In the 46 genomes sequenced, two clades, 19B/S/B4 and 20B, were the most represented (Figure 3(b)). The clade 19 A samples (Ghana/36523_S23/2020, Ghana/35183_S24/2020, and Ghana/2666_S32/2020) consistently had an unresolved nucleotide (Y) at position 8782 and 14,408, preventing their correct legacy clade assignments.

### Genomic variation

**Figure 4.**
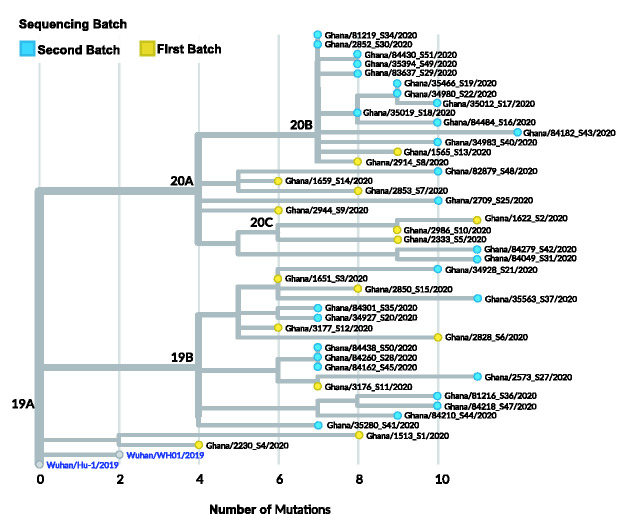
Divergence of the Ghanaian SARS-CoV-2 genomes relative to the Wuhan reference genome. The tree indicates the number of mutations in each of the isolates compared to the Wuhan/Hu-1/2019 reference genome. The viruses circulating in Ghana have 4–12 mutations. The maximum likelihood phylogenetic tree was constructed using IQ-TREE using the GTR substitution model, a clock rate of 0.0008, clock standard deviation of 0.0004, and mutations as the divergence unit. The 20A,B,C/G,GH,GR/A2a clade have accrued more mutations compared to the clade 19B/S/B4. Ghanaian samples have lower-level divergence than global samples but a similar level of D614G dominance. (A color version of this figure is available in the online journal.)

**Figure 5.**
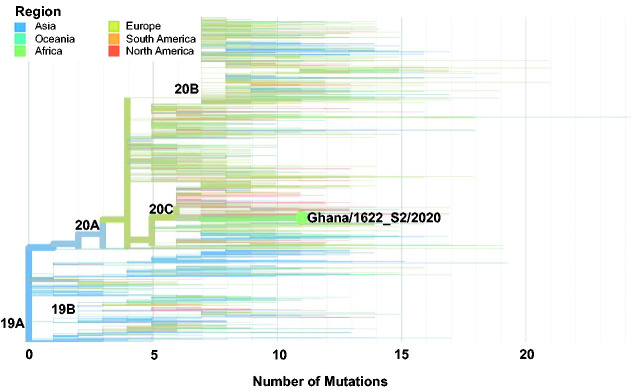
Maximum likelihood phylogenetic tree of global SARS-CoV-2 genomes depicting the Ghanaian sequence (Ghana/1622_S2/2020) with the highest mutations (*n* = 12). This sample was collected in March 2020 as an imported case from the United States of America, although the patient transited through the United Kingdom and Dubai. The mutational profile of this case clustered heavily with samples from all three regions: Europe, America, and Asia. The profile of the phylogenetic tree shows that majority of the Asian genomes cluster together and belong to clade 19B, while the African, European, and American genomes cluster together because they have evolved as subclades of 20A. Some genomes from Europe have acquired more than 25 mutations indicating a high level of divergence of isolates. (A color version of this figure is available in the online journal.)

The global mutational rate of SARS-CoV-2 is 2.5 nucleotides/month, but these SARS-CoV-2 genomes had lower than expected mutational rates, fewer mutations than would have been attributed to mutational rate of 2.5 (10 mutations by March and 15 mutations by June) (Figure 4). High rates of mutations usually correlate with high rates of transmission since mutations that persist tend to be important for viral adaptation. All (100%) of Ghanaian SARS-CoV-2 genomes had <13 mutations: 1 sequence had 12 mutations, 4 had 11 mutations, 10 had 10 mutations, and 34 had less than 9 mutations (Figure 4, Supplementary Table 3). Most genomes (86.6%) in the first batch had lower than nine mutations. However, the genome with the highest mutations (12), was Ghana/1622_S2/2020 one of the earliest imported cases in batch 1 (Figure 5). This isolate was from a patient who had arrived in the country from the United States and transited through the United Kingdom (UK) and Dubai. The mutational profile of this case clustered very closely with United States, Europe, and Asian samples (Figure 5). This patient exhibited severe disease at the time of sampling and was under ventilation, though currently, disease outcome is unknown. Another sample from the first batch, Ghana/2828_S6/2020, had 10 mutations. Both of these viruses were expected to be highly transmissible (based on mutational profile); however, these genotypes were not detected in the batch two samples (Supplementary Table 3).

As expected, a significant number of non-synonymous mutations (Supplementary Table 3) and synonymous mutations (variants) were detected (Supplementary Table 4). The spike variant D614G was present in 55% of the genomes (Supplementary Figure 4, Supplementary Table 3). Mutations in nucleocapsid, within the 202–205 amino acid motif, were found in >70% of genomes, ORF14 had variants in greater than 75% of genomes (predominated by G50E/R in A2a genomes and V49I in B4 genomes), and ORF1a had non-synonymous mutations in almost 50% of genomes, but these showed the most diversity of any of the genes/ORFs (Supplementary Table 3).

### Viral evolution

**Figure 6.**
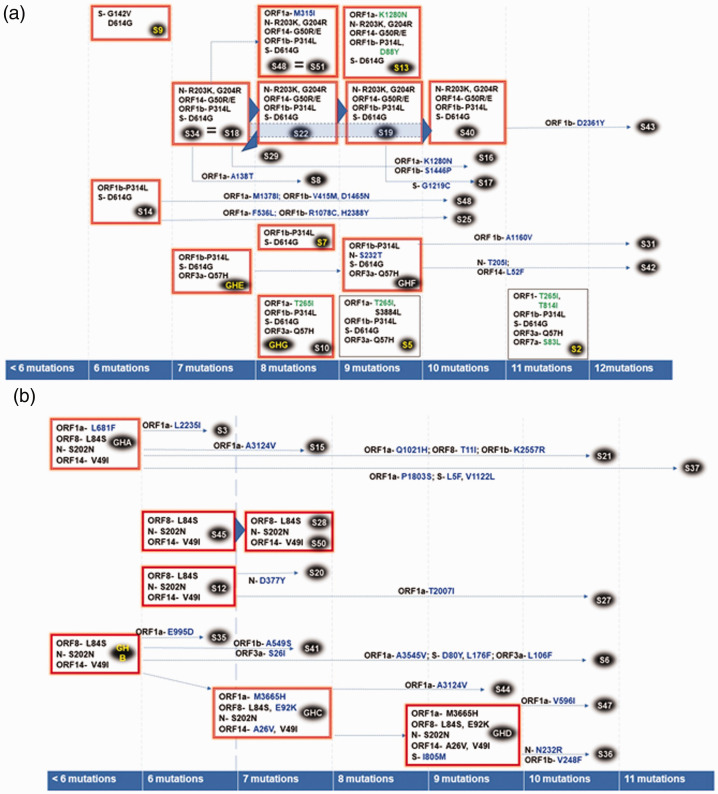
(a). D614G substitutions segregating to clade 20A,B,C/G,GH,GR/A2a. Relatedness was inferred based on genotypic and phenotypic sequence similarity and sequence of mutational appearance, along with available metadata on residential neighborhoods. Key: Major phenotypes are indicated in the manner “viral protein/locus- amino acid substitution”. Phenotypes are indicated in blue if they have only been reported in this study and show evidence of being locally evolved, green if they have not been reported elsewhere but are detected on an imported virus, and black if they have previously been reported elsewhere. “Major phenotypes/amino acid substitutions” (with the same genotype) are indicated in boxes; when that box is surrounded by a “red glow,” that virus has been transmitted. When silent mutations alone differentiate transmitted viruses, a thick blue arrow is used to link an antecedent virus with its descendent virus. A dotted line with an arrow links a virus with “major phenotype” to their transmitted descendants with additional phenotypic expression (indicated on top or below the dotted line). Sample IDs, indicated in small black ovals are colored yellow to indicate imported viruses and white to indicate locally acquired transmissions. Isolates with identical genotypes are linked using an equal (=) sign. Sample IDs prefaced by GH indicate undetected by deduced genotypes/phenotypes circulating in Ghana. Evolution and transmission analysis of the Ghanaian SARS-CoV-2 genomes harboring and (b). L84S substitutions segregating to clade 19B/S/B4. (A color version of this figure is available in the online journal.)

Genomes that clustered to 19B/S/B4 exhibited a high number of novel locally evolved mutations (Figure 6(b)) relative to genomes that clustered to 20 A,B,C/G,GH, GR/A2a (Figure 6(a)). For this study, we defined a locally evolved mutation as one that had not been previously reported elsewhere, or one which had been reported elsewhere, but was detected after one that had not previously been reported. Though we cannot rule them out, rare mutations that did not appear in more than one sequence were considered as evidence of continuing adaptation, but not as transmissible variants. Novel mutations were deemed to be part of a local transmission cluster if detected in more than one viral sequence. The 19B/S/B4 was common among patients in the first batch that had no recent travel history (4//9), suggesting that they were probably infected by one individual who had returned from a recent travel.

**Figure 7.**
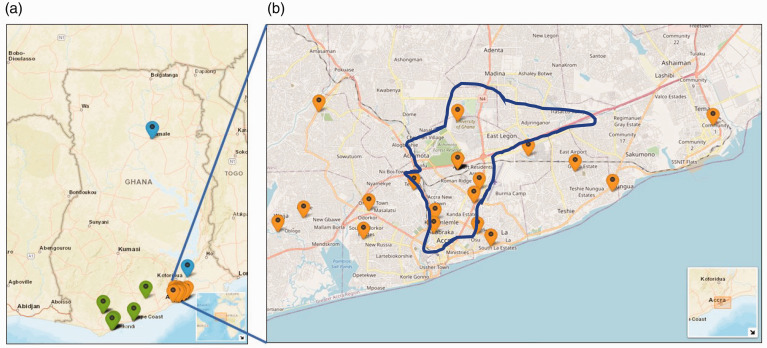
Map of Ghana showing the estimated physical residential coordinates of cases. (a) One of the genomes was isolated from northern Ghana (Tamale), while the majority of the cases were from the southern part of Ghana. There was a cluster of cases around Accra (capital city) and its environs. (b) The cases (8/46) that were reported in Ayawaso (Accra) and its surrounding areas. The residential addresses of the cases were used to generate the coordinates (latitude and longitude) using Google maps. The coordinates were plotted on a background world map using leaflet package in R software (Version 4.0.2). (A color version of this figure is available in the online journal.)

Two undetected genotypes (GHA and GHB) appear to have seeded the majority of 19B/S/B4 infections studied. Four genomes (from the Tema and Nungua municipalities of the Greater Accra Region as well as Sekondi-Takoradi in the Western Region) (Figure 7(a)) shared an L681F mutation in ORF1a. This indicates that an undetected transmissible variant (denoted GHA; Figure 6(b)) could be circulating and seeding infections in the coastal belt, i.e. Greater Accra, Central, and Western Regions.

Two other undetected variants GHC and GHD, appear to be seeding infections and radiating from the Ayawaso municipality of the Greater Accra Region (Figures 6(a) and 7(b)). In addition to the defining polymorphisms of 19B, GHC has a trifecta of non-synonymous mutations in ORF1a (M3665H), ORF8 (E92K), and ORF14 (A26V). This undetected virus is likely the parent of Ghana/84210_S44/2020; isolated from a resident of Ayawaso, Ghana/84218_S47/2020; isolated from another resident of Ayawaso and Ghana/81216_S36/2020; isolated from a resident of Cape Coast in the Central Region. The Ghana/84218_S47/2020 and Ghana/81216_S36/2020 viruses were likely seeded from an undetected descendant of GHC. GHD, which harbors the polymorphisms of GHC, as well as a novel spike amino acid substitution I805M, shared by both Ghana/84162_S6/2020, Ghana/81216_S36/2020, and Ghana/84162_S47/2020. The remaining 19B/S/B4 sequences appeared to indicate at least four sets of unevolved 19B/S/B4 seeding infections. Samples Ghana/84260_S28/2020 (from Ayawaso) and Ghana/84438_S50/2020 (from Odorkor Market, also in the Greater Accra region) appear to have been seeded by the same virus as sample Ghana/84162_S45/2020 (from Ayawaso). Sample Ghana/2828_S6/2020 (from East Cantonment, another suburb located in the Greater Accra Region) was one of the few highly evolved viruses sequenced in the first batch and showed some variations that have not been previously reported. The 20A, B, C/G, GR, GH/A2a genomes did not show extensive local evolution (Figure 6(a)). Five out of the six imported viruses sequenced clustered to these clades and most of the imported strains had been well evolved prior to introduction. As such, we did not discover evidence of as many transmitted non-synonymous polymorphisms. Importantly, Ghana/84049_S31/2020 (Ayawaso) and Ghana/84279_S42/2020 (Ayawaso) appear to have evolved from an undetected GHG viral genome, which harbors the S232T amino acid substitution in nucleocapsid. A series of 12 20B/GR/A2a genomes appeared to share genomes, multiple silent mutations, and a linkage to Ayawaso and various locations across southern Ghana.

## Discussion

By most accounts, SARS-CoV-2 mutates slowly, averaging about 2.5 mutations per month. There is evidence that mutations happen in quick succession, following long chronological stretches of genomic stability; this means most strains still resemble the Wuhan strain.^10,18^ Early on in the Chinese outbreak, there were reports of two possible variants of the virus referred to as the S (also referred to as the East Asian superclade B)^31^ and L lineages (also referred to as the European superclade A).^32^ The latter has been linked to greater transmissibility and pathogenicity, and currently the L lineage has diverged into the GISAID L, V, G, GH, and GR clades.^33,34^

In this study, by sequencing and phylogenetically characterizing SARS-CoV-2 viruses in Ghana, we have shown that locally circulating SARS-CoV-2 appears to exhibit a lower evolutionary rate than observed elsewhere.^11,31,35^ We have identified 17 genotypes with evidence of transmissible variants (∼9 variants). Most variants appear to have spread from the Greater Accra Region to the Central and Western regions of Ghana, with Ayawaso in East Legon as a likely epicenter. The locally sequenced SARS-CoV-2 viruses demonstrated very little variation from the original Wuhan strain, a common characteristic of this virus.^36^ Ten of the initial 15 viral genomes were closely linked to the European superclade A. Most genomes (8/15) clustered to 20 A, B, C/G, GR, GH/A2a; half of these had no history of travel, while the remaining genomes were from travelers from Hungary, India, USA (via Dubai) and the UK. Notably, all five of the batch 1 genomes that clustered to clade B4 had no evidence of travel or direct contact with a recent traveler, which is evidence of local transmission linked to the Asian superclade B. Though these initial genomes from March-April 2020 suggest ongoing local transmission in Ghana, the relative lack of variation suggested that most locally transmitted viruses were less than one month old, indicating a recent introduction and nascent expansion (late February/early March, 2020) of the virus into Ghana.^29^

We have identified a total of seven potential circulating genotypes of 19B/S/B4 as well as three apparently transmissible locally evolved genotypes GHA (ORF1a- L681F; ORF8- L84S; N- S202N; ORF14- V49I), GHC (ORF1a- M3665H; ORF8- L84S, E92K; N- S202N; ORF14- A26V, V49I), and GHD (ORF1a- M3665H; ORF8- L84S, E92K; N- S202N; ORF14- A26V, V49I; S- I805M). We have identified 12 likely circulating genotypes of Legacy clade A2a viruses. Our data show evidence of multiple importations of A2a viruses intoGhana, and apparent ongoing transmission within essential personnel in Accra (Greater Accra Region), Cape Coast (Central Region), Tarkwa and Sekondi-Takoradi (Western Region). Eleven genomes in our batch 2 sequencing round appeared to be linked; most were isolated from essential personnel in the Greater Accra, Central, and Western Regions. This suggests the patients might have contracted the infection from a single source early in the outbreak; likely during the lockdown. The Accra suburb of Ayawaso appears to feature prominently in linked genomes. This is unsurprising given that Ayawaso had been an infection hotspot early in the local outbreak, and remains so to date.

The D614G spike variant has spread across the world, dominating most genomes^37^ and constituting over 50% of genomes in this study. Although D614G has been implicated in increased transmissivity and higher mortality in other countries,^38–40^ available data from the Ghana Health Service do not appear to support this theory. Further investigations in a larger sample size will be necessary to clearly determine the impact of D614G on SARS-CoV-2 virulence in Ghana.

The potential impact of COVID-19 on Africa has long been discussed^41^ with most reports treating any impact on Africa uniformly. As at now, COVID-19 has impacted Africa in an uneven manner. Nine months after pandemic declaration, most African countries are reporting low numbers of cases, though this may be partly linked to low levels of testing uptake. Importantly even though, in countries with high levels of reliable testing, case fatality ratios (CFR) vary. Cote D'Ivoire (CFR ≈ 0.63%) and Ghana (CFR ≈ 0.65%) highlight countries with low CFR, whereas other countries such as, Kenya (CFR ≈ 1.75%), Nigeria (CFR ≈ 1.92%), and South Africa (CFR ≈ 2.42%) have higher CFRs.^2,42^ Several factors may contribute to the observed low CFRs in Africa, including younger population, cross-protection from existing immunity to other Coronaviruses, and early institution of safety protocols. In addition, local evolution of the virus as observed in this study, as well as others,^43^ may be driven by existing immunity to other pathogens and environmental factors that are unique to Africa. It would be interesting to investigate in future studies how these locally generated variants impact on transmissibility and virulence of the virus. In addition, in Ghana, the early border closures, quarantine measures, and lockdown may have limited the initial importation of SARSs-CoV-2 variants into the country leading to more favorable outcomes.

One isolate in our study, Ghana/2828_S6/2020, belonging to the legacy clade B4 was especially noteworthy. In that it exhibited four non-synonymous mutations; in ORF1a (A2745), ORF3a (L106F), and two spike mutations (D80Y and L176F). Though their roles are as yet to be elucidated, D80 and L176 are located on the outer edges of the S1 domain within the trimeric spike protein structure (PDB ID: 6VXX).^44,45^ D80 is located just after ß-sheet 4 and L176 is within the same ß-sheet domain. Interestingly, in the recently published cryo-EM structures, D80 is resolved in the closed state (PDB ID: 6VXX) but is not resolved in the open state (PDB ID: 6VYB). L176 is not resolved in either, but S172 is within 12 angstroms of D80. Given these observations, it would be interesting to look for these mutational combinations in subsequent sequences.^44^

This study provides some of the most in-depth genomic analysis of SARS-CoV-2 in Africa to date, and provides a framework for monitoring the evolution of the virus as it continues to spread on the continent and globally. Such studies are essential for tracking transmission dynamics and assessing the potential efficacy of any vaccines that become available in the future.

## Supplemental Material

sj-pdf-1-ebm-10.1177_1535370220975351 - Supplemental material for Genomic analysis of SARS-CoV-2 reveals local viral evolution in Ghana Click here for additional data file. Supplemental material, sj-pdf-1-ebm-10.1177_1535370220975351 for Genomic analysis of SARS-CoV-2 reveals local viral evolution in Ghana by Joyce M Ngoi, Peter K Quashie, Collins M Morang'a, Joseph HK Bonney, Dominic SY Amuzu, Selassie Kumordjie, Ivy A Asante, Evelyn Y Bonney, Miriam Eshun, Linda Boatemaa, Vanessa Magnusen, Erasmus N Kotey, Nicaise T Ndam, Frederick Tei-Maya, Augustina K Arjarquah, Evangeline Obodai, Isaac D Otchere, Yaw Bediako, Joe K Mutungi, Lucas N Amenga-Etego, John K Odoom, Abraham K Anang, George B Kyei, Bright Adu, William K Ampofo and Gordon A Awandare in Experimental Biology and Medicine
