# Supplementary material for: Genomic analysis of SARS-CoV-2 reveals local viral evolution in Ghana
Source: Exp Biol Med (Maywood). 2020 Dec 16;246(8):960–70. doi: 10.1177/1535370220975351 (PMC7746953; doi:10.1177/1535370220975351)
Supplement: sj-pdf-1-ebm-10.1177_1535370220975351 - Supplemental material for Genomic analysis of SARS-CoV-2 reveals local viral evolution in Ghana [file sj-pdf-1-ebm-10.1177_1535370220975351.pdf]

Supplementary Table 1 Demographics of study participants

Sample	Date of reporting	Place of sample collection	Temperature °C	Age	Sex	Travel history
Ghana/1513_S1/2020	3/23/2020	NA	NA	63	F	UK
Ghana/1622_S2/2020	3/24/2020	NA	36	42	M	USA, UK & Dubai
Ghana/1651_S3/2020	3/25/2020	NA	36	55	M	None
Ghana/2230_S4/2020	3/27/2020	NA	37.1	24	M	None
Ghana/2333_S5/2020	3/27/2020	NA	37	54	M	UK
Ghana/2828_S6/2020	3/29/2020	NA	37	38	M	None
Ghana/2853_S7/2020	3/29/2020	NA	36.4	53	F	Hungary
Ghana/2914_S8/2020	3/30/2020	NA	36.5	73	M	None
Ghana/2944_S9/2020	3/30/2020	NA	37	32	M	India
Ghana/2986_S10/2020	3/31/2020	NA	NA	60	M	None
Ghana/3176_S11/2020	3/30/2020	NA	37	28	F	None
Ghana/3177_S12/2020	3/30/2020	NA	36	30	F	None
Ghana/1565_S13/2020	3/24/2020	NA	36	27	M	Norway
Ghana/1659_S14/2020	3/25/2020	NA	36.5	32	F	None
Ghana/2850_S15/2020	3/29/2020	NA	NA	54	M	None
Ghana/84484_S16/2020	5/25/2020	Cape Coast Hospital	NA	21	F	NA
Ghana/35012_S17/2020	5/23/2020	37 Military Hospital	37	25	M	NA
Ghana/35019_S18/2020	5/23/2020	37 Military Hosp	36.6	30	M	NA
Ghana/35466_S19/2020	5/24/2020	Goldfields Hospital	36.5	NA	F	NA
Ghana/34927_S20/2020	5/23/2020	37 Military Hospital	37	25	M	NA
Ghana/34928_S21/2020	5/23/2020	37 Military Hospital	36.4	32	M	NA
Ghana/34980_S22/2020	5/23/2020	37 Military Hospital	36.7	28	M	NA
Ghana/36523_S23/2020	5/27/2020	BNI	36.7	NA	F	NA
Ghana/35183_S24/2020	5/24/2020	University of Ghana	36	4	M	NA
Ghana/2709_S25/2020	NA	NA	NA	NA	NA	NA
Ghana/2573_S27/2020	NA	NA	NA	NA	NA	NA
Ghana/84260_S28/2020	5/25/2020	Ayawaso East	NA	NA	F	NA
Ghana/83637_S29/2020	5/22/2020	Apruto Hospital	39.9	30	F	NA
Ghana/2852_S30/2020	NA	NA	NA	NA	NA	NA
Ghana/84049_S31/2020	5/25/2020	Ayawaso East	36.6	20	M	NA
Ghana/2666_S32/2020	NA	NA	NA	NA	NA	NA
Ghana/81219_S34/2020	5/20/2020	Cape Coast Hospital	36.2	43	F	NA
Ghana/84301_S35/2020	5/24/2020	Ayawaso	36.9	23	M	NA
Ghana/81216_S36/2020	5/20/2020	Cape Coast Hospital	NA	31	F	NA
Ghana/35563_S37/2020	NA	NA	NA	NA	NA	NA
Ghana/34983_S40/2020	5/23/2020	37 Military Hospital	36.9	43	F	NA
Ghana/35280_S41/2020	5/24/2020	37 Military Hospital	36.6	27	M	NA
Ghana/84279_S42/2020	5/25/2020	Ayawaso East	NA	NA	F	NA
Ghana/84142_S43/2020	5/25/2020	Ayawaso East	NA	NA	M	NA
Ghana/84210_S44/2020	5/24/2020	Ayawaso East	NA	30	M	NA
Ghana/84162_S45/2020	4/25/2020	Ayawaso East	NA	NA	F	NA
Ghana/84218_S47/2020	5/25/2020	Ayawaso East	NA	NA	M	NA
Ghana/82879_S48/2020	5/20/2020	Ablekuma North	NA	18	F	NA
Ghana/35394_S49/2020	5/24/2020	Goldfields Hospital	37.5	53	M	NA
Ghana/84438_S50/2020	5/24/2020	Ayawaso East	NA	NA	F	NA
Ghana/84430_S51/2020	5/25/2020	Brakwa Health Centre	36.5	33	M	NA

Supplementary Table 2: Clade analysis of each Ghanaian SARS-CoV-2 genomes

	Nextstrain clade	Legacy_ clades	Pangolin lineage	Deduced GISAID clades	Divergence/ Mutations	#Ns	#gaps
Ghana/2666_S32/2020	19A	unassigned	B.1.1	unassigned	0	593	0
Ghana/35183_S24/2020	19A	unassigned	B.1.5	unassigned	0	1014	0
Ghana/36523_S23/2020	19A	unassigned	B	unassigned	0	550	0
Ghana/2230_S4/2020	19A	unassigned	B.2.1	V	4	272	0
Ghana/1513_S1/2020	19A	unassigned	B.2.2	V	8	2829	0
Ghana/1651_S3/2020	19B	B4	A	S	6	302	0
Ghana/3177_S12/2020	19B	B4	A	S	6	889	0
Ghana/84162_S45/2020	19B	B4	A	S	6	709	27
Ghana/84301_S35/2020	19B	B4	A	S	6	1266	0
Ghana/3176_S11/2020	19B	B4	A	S	7	587	0
Ghana/34927_S20/2020	19B	B4	A	S	7	299	0
Ghana/35280_S41/2020	19B	B4	A	S	7	734	0
Ghana/84260_S28/2020	19B	B4	A	S	7	244	27
Ghana/84438_S50/2020	19B	B4	A	S	7	134	27
Ghana/2850_S15/2020	19B	B4	A	S	8	609	0
Ghana/84210_S44/2020	19B	B4	A	S	9	314	0
Ghana/2573_S27/2020	19B	B4	A	S	10	187	0
Ghana/2828_S6/2020	19B	B4	A	S	10	270	0
Ghana/34928_S21/2020	19B	B4	A	S	10	395	0
Ghana/81216_S36/2020	19B	B4	A	S	10	176	0
Ghana/84218_S47/2020	19B	B4	A	S	10	758	0
Ghana/35563_S37/2020	19B	B4	A	S	11	1	0
Ghana/1659_S14/2020	20A	A2a	B.1	G	6	272	0
Ghana/2944_S9/2020	20A	A2a	B.1	G	6	1449	0
Ghana/2853_S7/2020	20A	A2a	B.1	G	8	1346	0
Ghana/2709_S25/2020	20A	A2a	B.1.5	GH	10	18	10
Ghana/82879_S48/2020	20A	A2a	B.1	G	10	186	0
Ghana/84049_S31/2020	20A	A2a	B.1	G	11	69	0
Ghana/84279_S42/2020	20A	A2a	B.1	G	11	274	0
Ghana/2852_S30/2020	20B	A2a	B.1.1	GR	3	531	0
Ghana/35019_S18/2020	20B	A2a	B.1.1	GR	7	686	0
Ghana/81219_S34/2020	20B	A2a	B.1.1	GR	7	473	0
Ghana/2914_S8/2020	20B	A2a	B.1.1	GR	8	288	0
Ghana/34980_S22/2020	20B	A2a	B.1.1	GR	8	911	0
Ghana/35394_S49/2020	20B	A2a	B.1.1	GR	8	747	0
Ghana/83637_S29/2020	20B	A2a	B.1.1	GR	8	132	0
Ghana/84430_S51/2020	20B	A2a	B.1.1	GR	8	210	0
Ghana/1565_S13/2020	20B	A2a	B.1.1	GR	9	520	0
Ghana/35466_S19/2020	20B	A2a	B.1.1	GR	9	456	0
Ghana/34983_S40/2020	20B	A2a	B.1.1	GR	10	462	4
Ghana/35012_S17/2020	20B	A2a	B.1.1	GR	10	1	0
Ghana/84484_S16/2020	20B	A2a	B.1.1	GR	10	746	11
Ghana/84182_S43/2020	20B	A2a	B.1.1	GR	12	450	0
Ghana/2986_S10/2020	20C	A2a	B.1	GH	8	1890	0
Ghana/2333_S5/2020	20C	A2a	B.1.3	GH	9	239	0
Ghana/1622_S2/2020	20C	A2a	B.1	GH	11	389	0

#Ns – Number of missing nucleotides, #gaps - number of gaps

Supplementary Table 3: Analysis of each viral proteins and the type of mutations

	ORF1a	ORF8	N	ORF14	ORF1b	S	ORF3a	ORF7a
Ghana/2666_S32/2020								
Ghana/35183_S24/2020								
Ghana/36523_S23/2020								
Ghana/2230_S4/2020	I739V, P765S						G251V	
Ghana/1513_S1/2020	H417R				V2365F		G251V	
Ghana/1651_S3/2020	L681F, L2235I, N3833K	L84S	S202N	V49I				
Ghana/3177_S12/2020		L84S	S202N	V49I				
Ghana/84162_S45/2020		L84S	S202N	V49I				
Ghana/84301_S35/2020	E995D	L84S	S202N	V49I				
Ghana/3176_S11/2020		L84S	S202N	V49I				
Ghana/34927_S20/2020		L84S	S202N, D377Y	V49I				
Ghana/35280_S41/2020		L84S	S202N	V49I	A549S		S26L	
Ghana/84260_S28/2020		L84S	S202N	V49I				
Ghana/84438_S50/2020		L84S	S202N	V49I				
Ghana/2850_S15/2020	L681F, A3124V	L84S	S202N	V49I				
Ghana/84210_S44/2020	M3655I	L84S, E92K	S202N	A26V, V49I			S209T	
Ghana/2573_S27/2020	T2007I	L84S	S202N	V49I				
Ghana/2828_S6/2020	A2745V	L84S	S202N	V49I		D80Y, L176F	L106F	
Ghana/34928_S21/2020	L681F, Q1021H	T11I, L84S	S202N	V49I	K2557R			
Ghana/81216_S36/2020	M3655I	L84S, E92K	S202N, S232R	A26V, V49I	V248F	I805M		
Ghana/84218_S47/2020	V596I, M3655I	L84S, E92K	S202N	A26V, V49I		I805M		
Ghana/35563_S37/2020	L681F, P1803S	L84S	S202N	V49I		L5F, V1122L		
Ghana/1659_S14/2020					P314L	D614G		
Ghana/2944_S9/2020						G142V, D614G		
Ghana/2853_S7/2020					P314L	D614G		
Ghana/2709_S25/2020	F536L				P314L, R1078C, H2388Y	D614G		
Ghana/82879_S48/2020	M1378I				P314L, V415M, D1465N	D614G		
Ghana/84049_S31/2020			S232T		P314L, A1160V	D614G	Q57H	
Ghana/84279_S42/2020			T205I, S232T	L52F	P314L	D614G	Q57H	
Ghana/2852_S30/2020			R203K, G204R	G50R, G50E		D614G*		
Ghana/35019_S18/2020			R203K, G204R	G50R, G50E	P314L	D614G		
Ghana/81219_S34/2020			R203K, G204R	G50R, G50E	P314L	D614G		

Ghana/2914_S8/2020	A138T		R203K, G204R	G50R, G50E	P314L	D614G		
Ghana/34980_S22/2020			R203K, G204R	G50R, G50E	P314L	D614G		
Ghana/35394_S49/2020	M315I		R203K, G204R	G50R, G50E	P314L	D614G		
Ghana/83637_S29/2020			R203K, G204R	G50R, G50E	P314L	D614G		
Ghana/84430_S51/2020	M315I		R203K, G204R	G50R, G50E	P314L	D614G		
Ghana/1565_S13/2020	K1230N		R203K, G204R	G50R, G50E	P314L, D884Y	D614G		
Ghana/35466_S19/2020			R203K, G204R	G50R, G50E	P314L	D614G		
Ghana/34983_S40/2020			R203K, G204R	G50R, G50E	P314L	D614G		
Ghana/35012_S17/2020			R203K, G204R	G50R, G50E	P314L	D614G, G1219C		
Ghana/84484_S16/2020	K1280N		R203K, G204R	G50R, G50E	P314L, S1446P	D614G		
Ghana/84182_S43/2020			R203K, G204R	G50R, G50E	P314L, D2361Y	D614G		
Ghana/2986_S10/2020	T265I				P314L	D614G	Q57H	
Ghana/2333_S5/2020	T265I, S3884L				P314L, A1844V	D614G	Q57H	
Ghana/1622_S2/2020	T265I, T814I				P314L, M2462I	D614G	Q57H	S83L

8

Supplementary Table 4: Nucleotide mutational analysis of Ghanaian SARS-CoV-2 genomes.

Sample ID	excess divergence	Nucleotide mutations
Ghana/1513_S1/2020	0.19	A1515G,C9223T,C14805T,T17247C,A17799G,G20560T,G26144T,G29688T
Ghana/2986_S10/2020	-0.29	C241T,C1059T,C3037T,C4321T,C6040T,C14408T,A23403G,G25563T
Ghana/3176_S11/2020	-1.22	A361G,C2062T,C8782T,G22468T,T28144C,G28878A,G29742A
Ghana/3177_S12/2020	-2.22	C8782T,C21844T,C24370T,T28144C,G28878A,G29742A
Ghana/1565_S13/2020	1.19	C241T,C3037T,G3955T,C14408T,G16117T,A23403G,G28881A,G28882A,G28883C
Ghana/1659_S14/2020	-1.88	C241T,C3037T,T10561C,C14408T,C15324T,A23403G
Ghana/2850_S15/2020	-0.15	C2306T,C8782T,C9636T,T14382C,C24370T,T28144C,G28878A,G29742A
Ghana/84484_S16/2020	-3.97	C241T,C745T,C3037T,G4105T,C14408T,T17803C,A23403G,G28881A,G28882A,G28883C
Ghana/35012_S17/2020	-3.97	C241T,C745T,C3037T,A5401G,C14408T,A23403G,G25217T,G28881A,G28882A,G28883C
Ghana/35019_S18/2020	-6.97	C241T,C3037T,C14408T,A23403G,G28881A,G28882A,G28883C
Ghana/35466_S19/2020	-4.97	C241T,C745T,C3037T,A5401G,C14408T,A23403G,G28881A,G28882A,G28883C
Ghana/1622_S2/2020	3.19	C241T,C1059T,C2706T,C3037T,C4321T,C6040T,C14408T,G20853T,A23403G,G25563T,C28265T
Ghana/34927_S20/2020	-6.97	C8782T,C24370T,C27641T,T28144C,G28878A,G29402T,G29742A
Ghana/34928_S21/2020	-3.97	G242A,C2306T,G3328T,C8782T,A21137G,C24370T,C27925T,T28144C,G28878A,G29742A
Ghana/34980_S22/2020	-5.97	C241T,C745T,C3037T,C14408T,A23403G,G28881A,G28882A,G28883C
Ghana/36523_S23/2020	-12.19	
Ghana/35183_S24/2020	-11.99	
Ghana/2709_S25/2020	-3.97	C241T,T1873G,C3037T,C14408T,C16699T,A20268G,C20629T,A23403G,C27211A,G29734C
Ghana/2573_S27/2020	-3.97	A361G,C2062T,C6285T,G8179T,C8782T,C11747T,C27615T,T28144C,G28878A,G29742A
Ghana/84260_S28/2020	-6.97	A361G,C829T,C8782T,G22468T,T28144C,G28878A,G29742A
Ghana/83637_S29/2020	-5.97	C241T,C3037T,C14408T,G21138A,A23403G,G28881A,G28882A,G28883C
Ghana/1651_S3/2020	-1.88	C2306T,C8782T,C24370T,T28144C,G28878A,G29742A
Ghana/2852_S30/2020	-10.97	G28881A,G28882A,G28883C
Ghana/84049_S31/2020	-2.97	C241T,C3037T,C14408T,C16946T,C18687T,C18877T,A23403G,G25563T,C28708T,G28968C,C28969T
Ghana/2666_S32/2020	-12.19	
Ghana/81219_S34/2020	-6.97	C241T,C3037T,C14408T,A23403G,G28881A,G28882A,G28883C
Ghana/84301_S35/2020	-7.97	G3250T,C8782T,C24370T,T28144C,G28878A,G29742A
Ghana/81216_S36/2020	-3.97	C8782T,G11230T,G14209T,A23977G,T28144C,G28167A,C28810T,G28878A,C28969A,G29742A
Ghana/35563_S37/2020	-2.97	C2306T,C5672T,C5986T,C8782T,C13515T,C21575T,C24370T,G24926T,T28144C,G28878A,G29742A
Ghana/2230_S4/2020	-3.88	A2480G,C2558T,C14805T,G26144T
Ghana/34983_S40/2020	-3.97	C241T,C3037T,C11620T,C14408T,C17634T,T22084C,A23403G,G28881A,G28882A,G28883C
Ghana/35280_S41/2020	-6.97	C8782T,G15112T,C25469T,T28144C,G28166A,G28878A,G29742A

Ghana/84279_S42/2020	-2.97	C241T,C3037T,C14408T,T14454C,C18687T,C18877T,A23403G,G25563T,C28887T,G28968C,C28969T
Ghana/84142_S43/2020	-1.97	C119T,C241T,C3037T,C14408T,G20548T,A23403G,G26211T,C26534T,G28881A,G28882A,G28883C,G29543C
Ghana/84210_S44/2020	-4.97	C8782T,G11230T,T26017A,T28144C,G28167A,C28810T,G28878A,G29742A,G29853A
Ghana/84162_S45/2020	-7.97	A361G,C829T,C8782T,T28144C,G28878A,G29742A
Ghana/84218_S47/2020	-3.97	G2051A,C8782T,G11230T,A23977G,T26876C,T28144C,G28167A,C28810T,G28878A,G29742A
Ghana/82879_S48/2020	-3.97	C241T,C3037T,T3547C,G4399T,C14408T,G14710A,C15324T,G17860A,A23403G,A23416T
Ghana/35594_S49/2020	-5.97	C241T,G1210T,C3037T,C14408T,A23403G,G28881A,G28882A,G28883C
Ghana/2333_S5/2020	0.99	C241T,C1059T,C3037T,C11916T,C14408T,C18998T,A23403G,G25563T,G29540A
Ghana/84438_S50/2020	-6.97	A361G,C829T,C8782T,G22468T,T28144C,G28878A,G29742A
Ghana/84430_S51/2020	-5.97	C241T,G1210T,C3037T,C14408T,A23403G,G28881A,G28882A,G28883C
Ghana/2828_S6/2020	1.85	C745T,C8499T,C8782T,G21800T,C22088T,C24370T,C25708T,T28144C,G28878A,G29742A
Ghana/2853_S7/2020	-0.15	C241T,C3037T,C14408T,C15324T,C18570T,A23403G,A23665C,T24769G
Ghana/2914_S8/2020	-0.22	C241T,G677A,C3037T,C14408T,A23403G,G28881A,G28882A,G28883C
Ghana/2944_S9/2020	-2.22	A187G,C241T,C3037T,C14408T,G21987T,A23403G

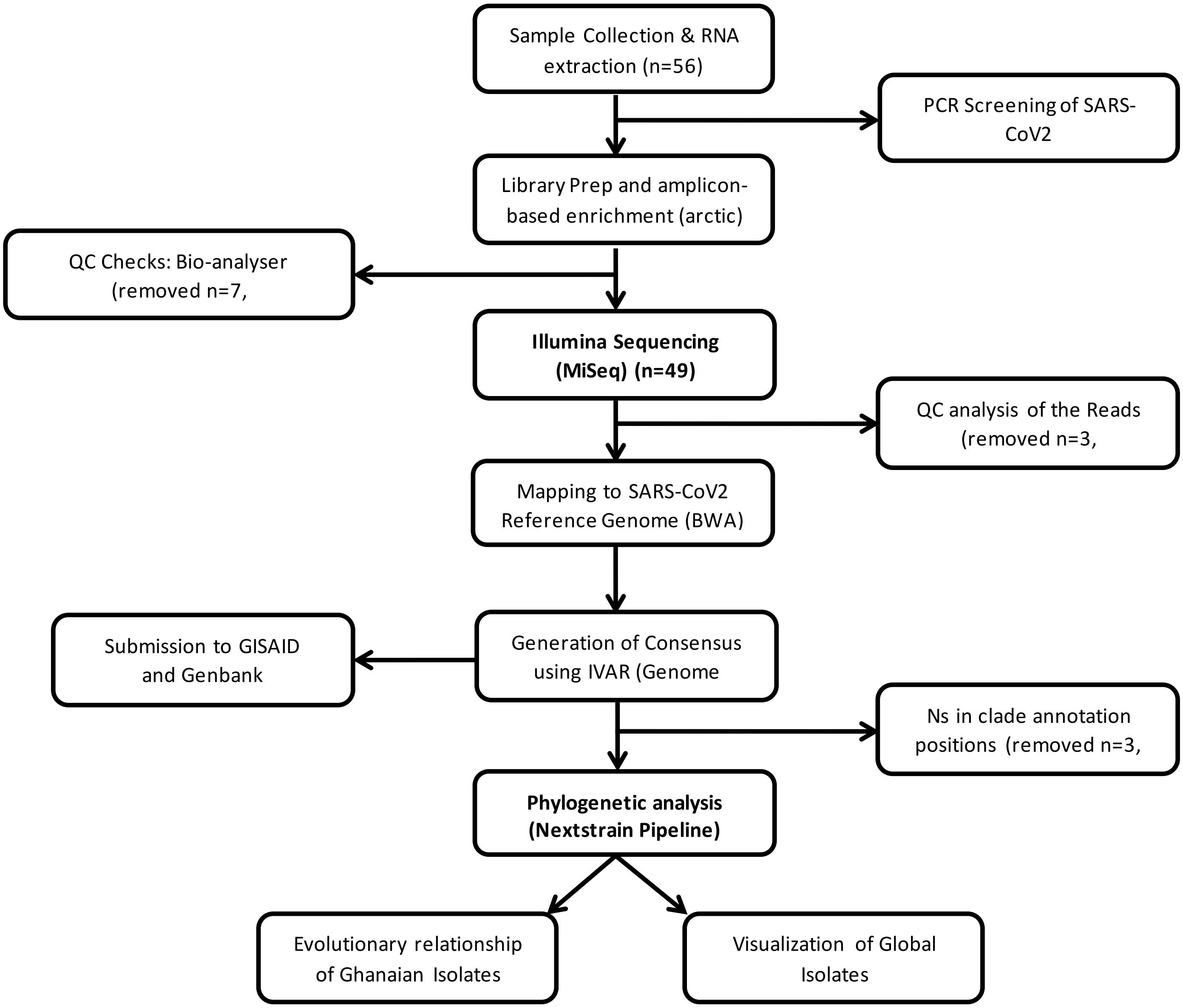

Supplementary Figure 1. Flowchart depicting workflow from sample acquisition to phylogenetic analysis. Fifty-six (56) COVID-19 samples were collected under a National Epidemic response directive. Viral RNA was extracted from as SARS-COV-2 positive by RT-PCR (Ct =18-25) nasopharyngeal and oropharyngeal samples. Viral cDNA was prepared, subjected to a multiplex PCR using the ARTIC nCoV-2019 /V1/V3 primers, sequencing libraries prepared using the Nextera XT DNA Library Preparation Kit and the Nextera XT Index Kit v2 Set

A (Illumina). Library quality control was carried out using the Agilent 2100 Bioanalyzer (Agilent) (seven samples were removed), and sequenced on the Illumina MiSeq system using the MiSeq® Reagent Kit v3 600 cycle. We removed 3 reads after read quality QC. Reads were trimmed using trim galore (Version 0.6.5) and mapped using BWA (version 0.7.17) to SARS-CoV-2 reference genome (https://github.com/connor-lab/ncov2019-artic-nf). Ivar was used for primer trimming (arctic primers V3), variant calling and consensus. Three (3) assembled genomes were removed from further phylogenetic analysis after being subjected to final QC for clade assignments. We performed phylogenetic analysis on SARS-CoV-2 genomic data using Nextstrain pipelines (https://github.com/nextstrain/ncov).

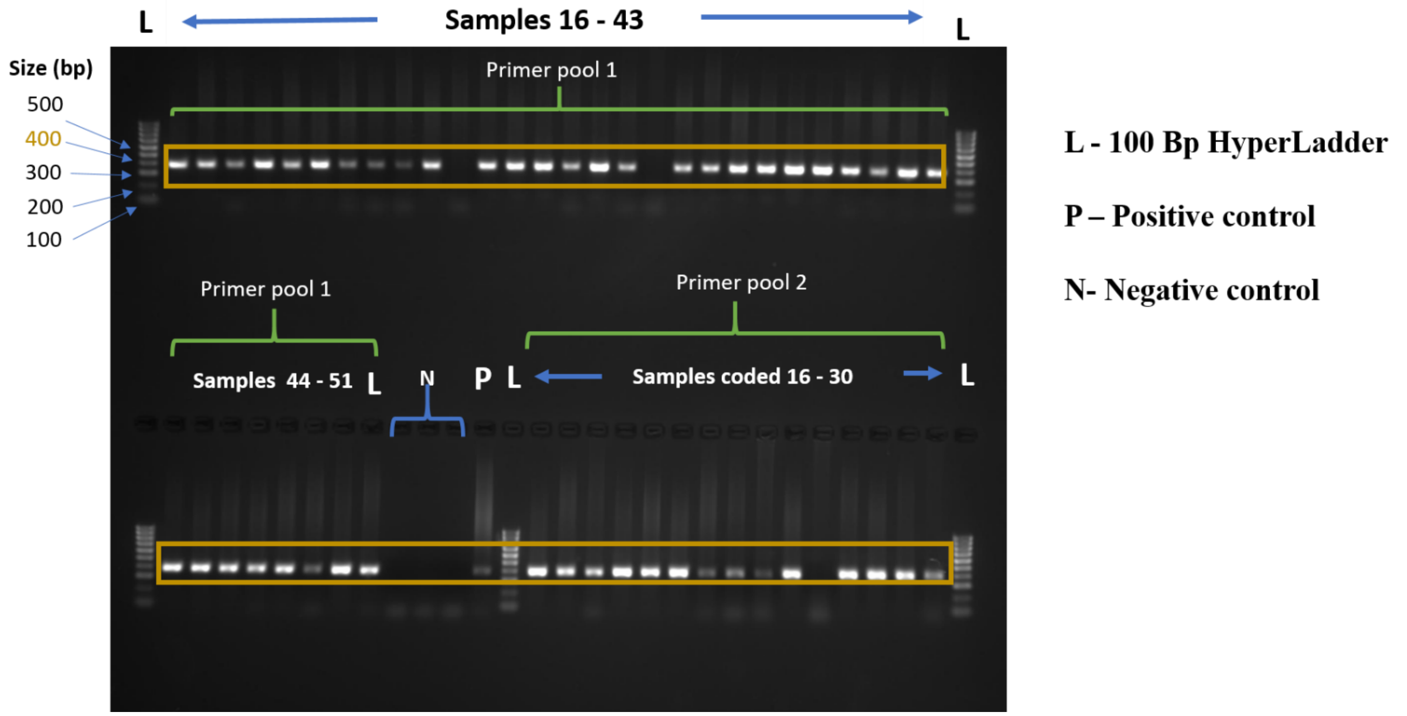

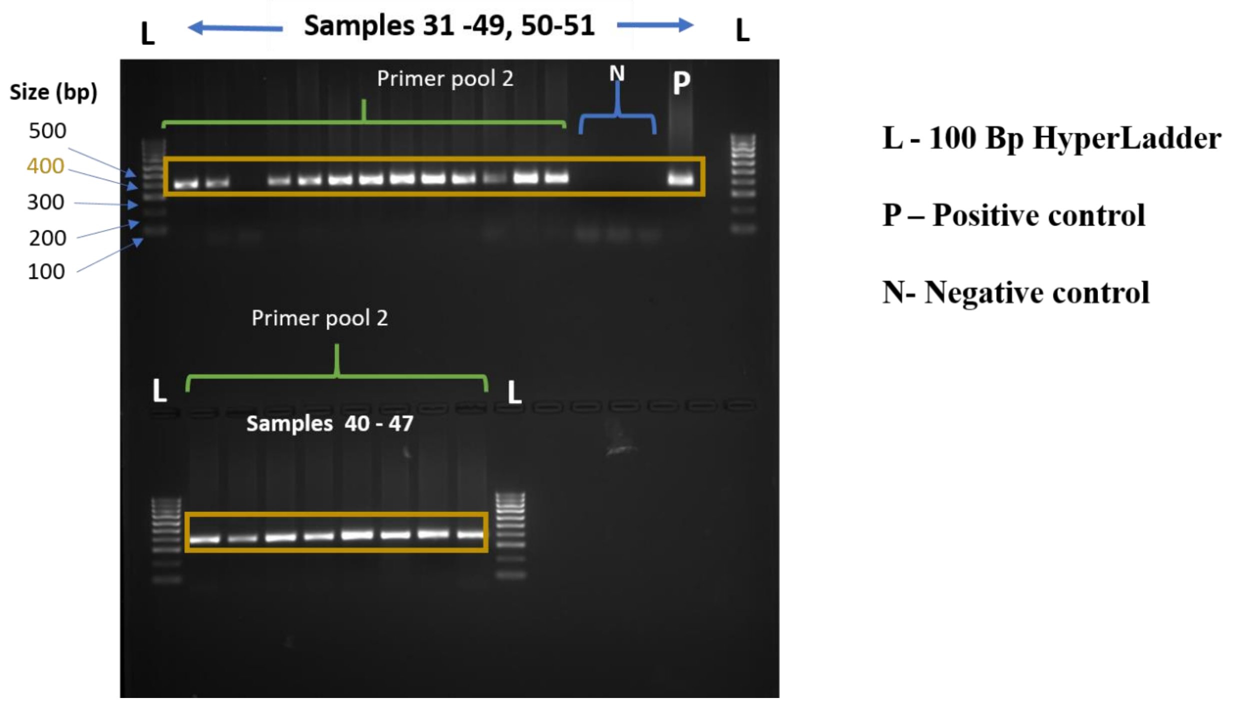

Supplementary Figure 2: Gel Image for batch2 PCR products

Supplementary Figure 3

Supplementary Figure 3: Phylogenetic tree showing country of exposure/Travel history/origin Ghanaian SARS-Cov-2 Viruses.

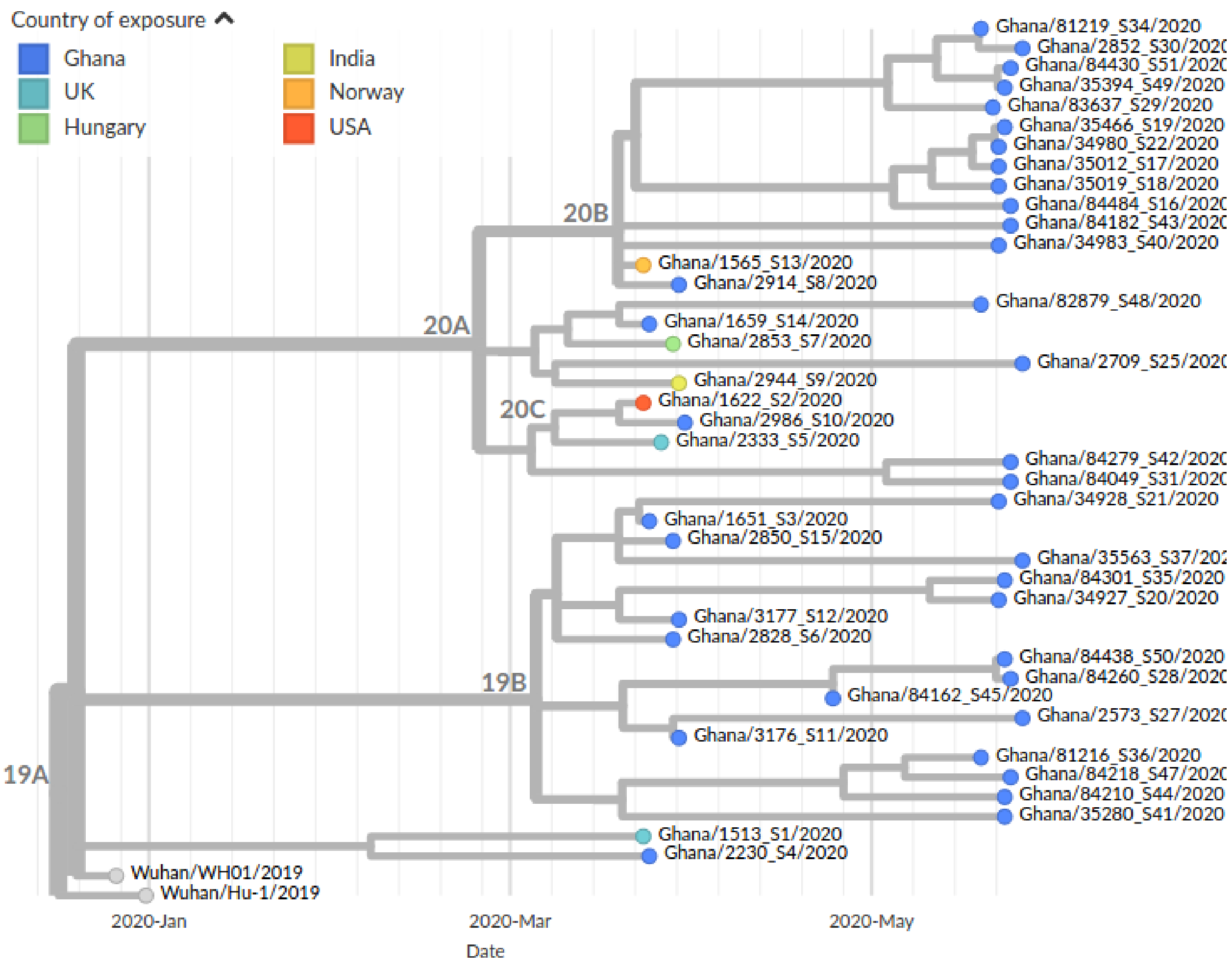

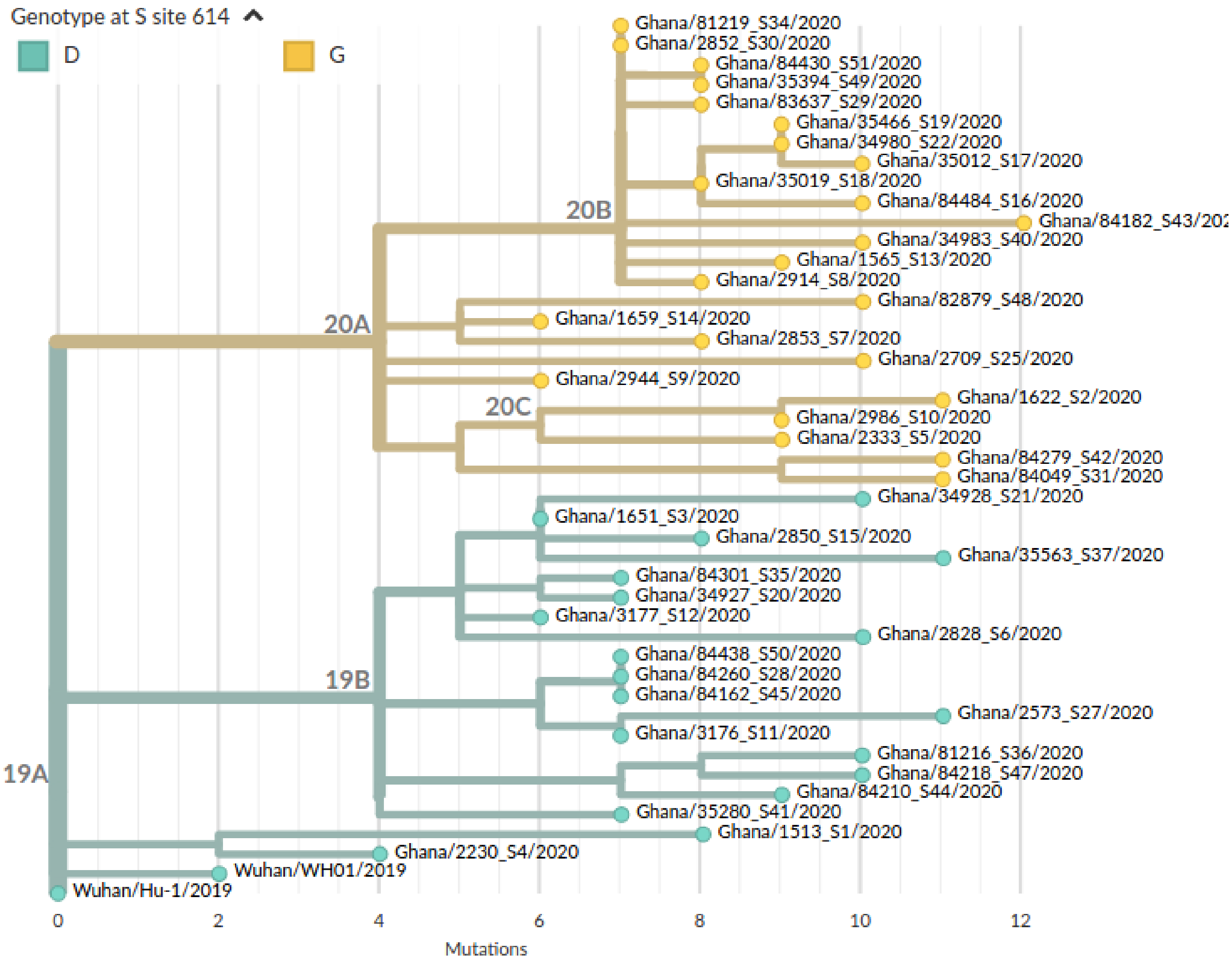

Supplementary Figure 4: Phylogenetic tree showing the Ghanaian SARS-Cov-2 genomes with D614G mutation Virus
